# Poor Eating Habits and Selected Determinants of Food Choice Were Associated With Ultraprocessed Food Consumption in Brazilian Women During the COVID-19 Pandemic

**DOI:** 10.3389/fnut.2021.672372

**Published:** 2021-05-13

**Authors:** Fabiana Infante Smaira, Bruna Caruso Mazzolani, Gabriel Perri Esteves, Heloisa C. Santo André, Milla Cordeiro Amarante, Daniela Fernandes Castanho, Karen Jennifer de Campos, Fabiana Braga Benatti, Ana Jéssica Pinto, Hamilton Roschel, Bruno Gualano, Carolina Ferreira Nicoletti

**Affiliations:** ^1^Applied Physiology & Nutrition Research Group, Rheumatology Division, School of Physical Education and Sport, Faculdade de Medicina FMUSP, Universidade de São Paulo, São Paulo, Brazil; ^2^Laboratory of Assessment and Conditioning in Rhematology, Faculdade de Medicina FMUSP, Disciplina de Reumatologia, Universidade de São Paulo, São Paulo, Brazil; ^3^School of Applied Sciences, State University of Campinas, Limeira, Brazil; ^4^Food Research Center, University of São Paulo, São Paulo, Brazil

**Keywords:** eating behavior, food processing level, SARS-CoV-2, quarantine, macronutrient intake

## Abstract

**Background:** The aim of this study was to investigate possible associations between food consumption and eating habits and food choice determinants in women during COVID-19 pandemic.

**Methods:** This is a cross-sectional survey conducted in Brazil between June and September, 2020, during which time social distancing measures were in place.

**Results:** Participants (*n* = 629) were aged 34.0 years and mostly within normal weight according to BMI (60.4%). “Snacking” and “liking” associated with increased energy (β = 164.27 and β = 110.24) and carbohydrate intake (β = 1.97 and β = 1.80), and with reduced protein intake (β = −1.54 and β = −1.18). In contrast, “dieting” and “weight control” associated with reduced energy (β = −162.57 and β = −111.49) and carbohydrate intake (β = −2.78 and β = −2.07), and with increased protein intake (β = 3.78 and β = 1.65). “Dieting” (β = 7.27), “need and hunger” (β = 3.34), and “health” (β = 4.94) associated with an increased consumption of unprocessed and minimally processed foods, whereas “replacing main meals with snacks” (β = −8.98), “snacking” (β = −6.92) and binge eating symptoms (β = −0.34) associated with reduced consumption of foods within this processing level. In contrast, “use of delivery services” (β = 3.39), “replacing main meals with snacks” (β = 5.49), “visual appeal” (β = 2.17), “social norms” (β = 2.19) and “affect regulation” (β = 2.01) associated with increased ultraprocessed food consumption. Overall, associations were more frequent and pronounced when analyzing food consumption by processing level rather than by macronutrient intake.

**Conclusion:** Some eating habits and food choice determinants (“snacking,” “replacing meals with snacks,” “use of delivery services”) observed during the COVID-19 pandemic were associated with an unhealthy diet (high energy and carbohydrate consumption, increased ultraprocessed food consumption and reduced unprocessed/minimally processed foods consumption) in Brazilian women.

## Introduction

The determinants of food consumption are factors that affect the choice of food through individual thoughts and feelings, and refer to why and how people eat, which foods they eat, and with whom they eat, as well as the ways people obtain, store, use, and discard food (1–3), resulting in actions or behaviors toward food consumption (i.e., eating habits) (4). As decisions related to eating habits and food choices are performed daily and in similar contexts, they likely result from a habitual response (5, 6), which, despite being relatively stable during adulthood (7), are prone to variation due to changes in daily routine and environment (8).

Social distancing measures necessary to contain the spread of SARS-CoV-2, changed lifestyle behaviors across the globe, including eating habits (9–12). In fact, unhealthier eating habits and food choices have been reported during the COVID-19 pandemic, such as overeating, snacking, replacing main meals with snacks, increased use of delivery services, and high ultraprocessed food intake (12–16). In a recent publication (17), we reported that Brazilian women increased eating habits such as cooking, use of delivery services, eating at the table, and snacking, during the COVID-19 pandemic, whereas they decreased participation in grocery shopping and dieting. Nonetheless, these findings are not unanimous, and some positive changes have also been observed, such as a rise in frequency of cooking, mirrored by an increase in consumption of home-cooked meals (13, 18). Importantly, it is currently unclear how these mixed changes in eating habits and food choices associate with food consumption during the COVID-19 pandemic. Exploring these aspects is key to understanding which changes may be potentially beneficial or detrimental to overall health.

The current analysis aimed to investigate possible associations between eating habits and food choice determinants with macronutrient intake and food consumption by processing level. We hypothesized that changes in eating habits and food choice determinants would have both positive and negative influences on selected aspects of food consumption (i.e., macronutrient intake and food processing level).

## Methods

### Study Design and Participants

This is an exploratory analysis comprising a subset of participants from a larger cross-sectional survey (17), conducted between June and September, 2020, a period in which a set of social distancing measures intended to contain the spread of COVID-19 were in place in Brazil. Considering the number of researchers and working capacity of our team, a subsample (*n* = 629) of participants from our original study (*n* = 1,183) (14) was randomly selected with the aim of analyzing 1-day food diaries in order to determine macronutrient intake and food consumption by processing level. Data from the survey were reported on a previous manuscript (14), except for the 1-day food diary.

Participants were recruited through advertisements on social media platforms (Facebook®, WhatsApp®, Instagram®, and Twitter®), press release, television, and radio. Inclusion criteria were as follows: women aged ≥18 years, currently living in Brazil, with ability to read, and with internet access.

All participants completed an online survey using the Google® Forms platform (Google® LLC, Menlo Park, CA, USA). Data related to their demographic, socioeconomic, and anthropometric characteristics, eating habits, food choice determinants, psychological symptoms, and food consumption were obtained.

This study was approved by the local ethical committee and was conducted in accordance with the Helsinki declaration. An approved Informed Consent Form was signed digitally by all participants before initiating the survey.

### Evaluation Tool

The online survey included questions about age, ethnicity, marital status, educational level, smoking, and chronic medical conditions, as well as anthropometric data [i.e., self-reported weight and height, which was then used to calculate body mass index (BMI)], eating habits, food choice determinants, eating attitudes, psychological symptoms, and food consumption.

### Outcomes

Macronutrient intake and food consumption by processing level were assessed using a 1-day food diary. Participants were instructed to fully report the quantity and type of foods and beverages that they consumed within the previous 24 h. Participants were provided with a food diary template. Analysis was then performed using the Dietbox software (online version). Food preparations (e.g., soups, puree, pies, sandwiches) were broken down into foods and ingredients, according to standardized recipes. Total energy intake (kcal) and macronutrient intake [grams and percentage of total energy intake (%TEI)] were calculated. Energy contribution (%TEI) and frequency of food consumption (times/day) were calculated for each processing level, according to the NOVA classification, and within the following categories: (i) unprocessed and minimally processed foods, such as edible parts of plants or animals, or natural foods altered by simple processes, such as fruits, vegetables, meat, egg, milk, but without adding substances, such as salt, sugar or fat; (ii) culinary ingredients, such as salt, sugar, butter or vegetable oils; (iii) processed foods, such as canned vegetables and fruits, salted nuts and seeds, cured or smoked meats; cheeses and unpackaged freshly made bread; (iv) ultraprocessed foods, such as carbonated drinks, ice-cream, cookies, pre-prepared pasta, pie or pizza, hot dogs, burgers, or instant soup noodles (19).

Eating habits (i.e., “participation in grocery shopping,” “cooking,” “use of delivery services,” “replacing main meals with snacks,” “eating at the table,” “eating in front of television/tablet/cellphone,” “snacking,” and “dieting”) were classified as binary outcomes (i.e., “yes,” if participant reported certain eating habit, or “no,” if participant reported the absence of certain eating habit). Food choice determinants (i.e., “liking,” “habits,” “need and hunger,” “health,” “convenience,” “pleasure,” “traditional eating,” “natural concerns,” “sociability,” “price,” “visual appeal,” “weight control,” “social norms,” “social image,” and “affect regulation”) were assessed by The Eating Motivation Survey (TEMS) (20), which comprises 45 questions preceded by “I eat what I eat,…”. The answers are given in a seven-point scale ranging from 1 (“never”) to 5 (“always”), with higher scores representing a higher impact of a given food choice determinant. Eating attitudes were assessed by the Binge Eating Scale (BES), which evaluates symptoms of binge eating episodes (21, 22), and by the Disordered Eating Attitude Scale (DEAS), which evaluates eating attitudes (23, 24). Higher scores represent more symptoms of binge eating episodes (BES score range: 0–46), and more dysfunctional eating attitudes (DEAS score range: 17–75). Psychological symptoms (i.e., depression, anxiety and stress symptoms, and loneliness) were assessed by Depression Anxiety Stress Scale-21 (DAS-21) (25, 26) and by the UCLA Loneliness Scale (UCLA-LS) (27, 28), with higher scores representing more symptoms (DAS-21 score range for: 0–28; UCLA-LS score range for: 1–8).

### Statistical Analysis

Descriptive data are presented as mean ±95% confidence interval (95% CI) for continuous variables and absolute and relative frequency (*n* [%]) for categorical variables. The association (linear regression) between eating habits/food choice determinants with macronutrient intake and with processing level were also tested, assuming eating habits/food choice determinants (e.g., “cooking,” “snacking,” “liking”) as independent variables, and macronutrient intake and processing level (e.g., energy, carbohydrate and unprocessed and minimally processed food consumption) as dependent variables. Additionally, we also tested the association between macronutrient intake and food consumption by processing level and was tested using linear regression models, assuming processing level (e.g., unprocessed and minimally processed, processed, and ultraprocessed food consumption) as independent variables, and macronutrients (e.g., carbohydrate, protein and fat intake) as dependent variables. All regression models were adjusted for age, BMI, educational level, ethnicity, marital status, and number of comorbidities. Data are presented as β (95% CI). All analyses were performed using the statistical package SAS (version 9.4). The level of significance was set at *p* ≤ 0.05.

## Results

Out of 1,183 women who participated in the original study (17), 629 were randomly selected and were included in this analysis. Among them, 129 participants did not properly report portion sizes, and their data regarding macronutrient intake and energy contribution were not included. Participant's age ranged from 18 to 72 years (34.0 [95% CI: 33.0, 35.0] years) and most participants were within normal weight according to BMI (60.4%), white (79.3%), single (56.9%), and had a high educational level (69.8%). Subsample characteristics were not different from the total sample (Table 1).

**Table 1:** General characteristics of participants.

	**Total** ** (*n* = 1,183)**	**Sub-sample** ** (*n* = 629)**	***p***
Age (years)	34.6 (33.9, 35.3)	34.0 (33.0, 35.0)	0.356
BMI (kg/m^2^)	24.8 (24.5, 25.1)	24.8 (24.4, 25.2)	0.963
**Self-related ethnicity**			
White	921 (77.8%)	497 (79.3%)	0.560
Yellow	41 (3.5%)	26 (4.2%)	
Brown	159 (13.4%)	76 (12.1%)	
Black	51 (4.3%)	25 (4.0%)	
Indigenous	6 (0.5%)	3 (0.5%)	
**Marital status**			
Married	436 (36.9%)	223 (35.5%)	0.629
Single	657 (55.5%)	357 (56.9%)	
Divorced	78 (6.6%)	43 (6.9%)	
Widow	12 (1.0%)	5 (0.8%)	
**Educational level**			
Elementary school	12 (0.1%)	6 (1.0%)	0.225
High school degree	314 (26.5%)	181 (28.8%)	
University degree	857 (72.4%)	438 (69.8%)	
**Presence of chronic diseases**			
Hypertension	50 (4.2%)	22 (3.5%)	0.454
Diabetes mellitus	17 (1.4%)	10 (1.6%)	0.795
Dyslipidemia	77 (6.5%)	40 (6.4%)	0.909
Thyroid disorders	109 (9.2%)	60 (9.6%)	0.375
Cardiovascular diseases	21 (1.8%)	15 (2.4%)	0.375
**Smoking habit**			
No	1,120 (94.8%)	600 (95.5%)	0.422
Yes	63 (5.3%)	28 (4.5%)	

*Data presented as mean and 95% confidence interval (95% CI) or absolute and relative frequency (n[%]).BMI, body mass index*.

Linear regression models showed “snacking” and “liking” associated with increased energy (β = 164.27 [61.09, 267.46], *p* = 0.002 and β = 110.24 [37.67, 182.81], *p* = 0.003) and carbohydrate intake (β = 1.97 [0.15, 3.78], *p* = 0.033 and β = 1.80 [0.53, 3.07], *p* = 0.005), and with reduced protein intake (β = −1.54 [−2.88, −0.21], *p* = 0.023 and β = −1.18 [−2.12, −0.24], *p* = 0.013). In contrast, “dieting” and “weight control” were associated with reduced energy (β = −162.57 [−289.49, −35.66], *p* = 0.012 and β = −111.49 [−170.46, −52.52], *p* < 0.001) and carbohydrate intake (β = −2.78 [−5.00, −0.56], *p* = 0.014 and β = −2.07 [−3.10, −1.04], *p* < 0.0001), and with increased protein intake (β = 3.78 [2.17, 5.39], *p* < 0.0001 and β = 1.65 [0.89, 2.40], *p* < 0.0001) (**Figure 2**).

**Figure 1:**
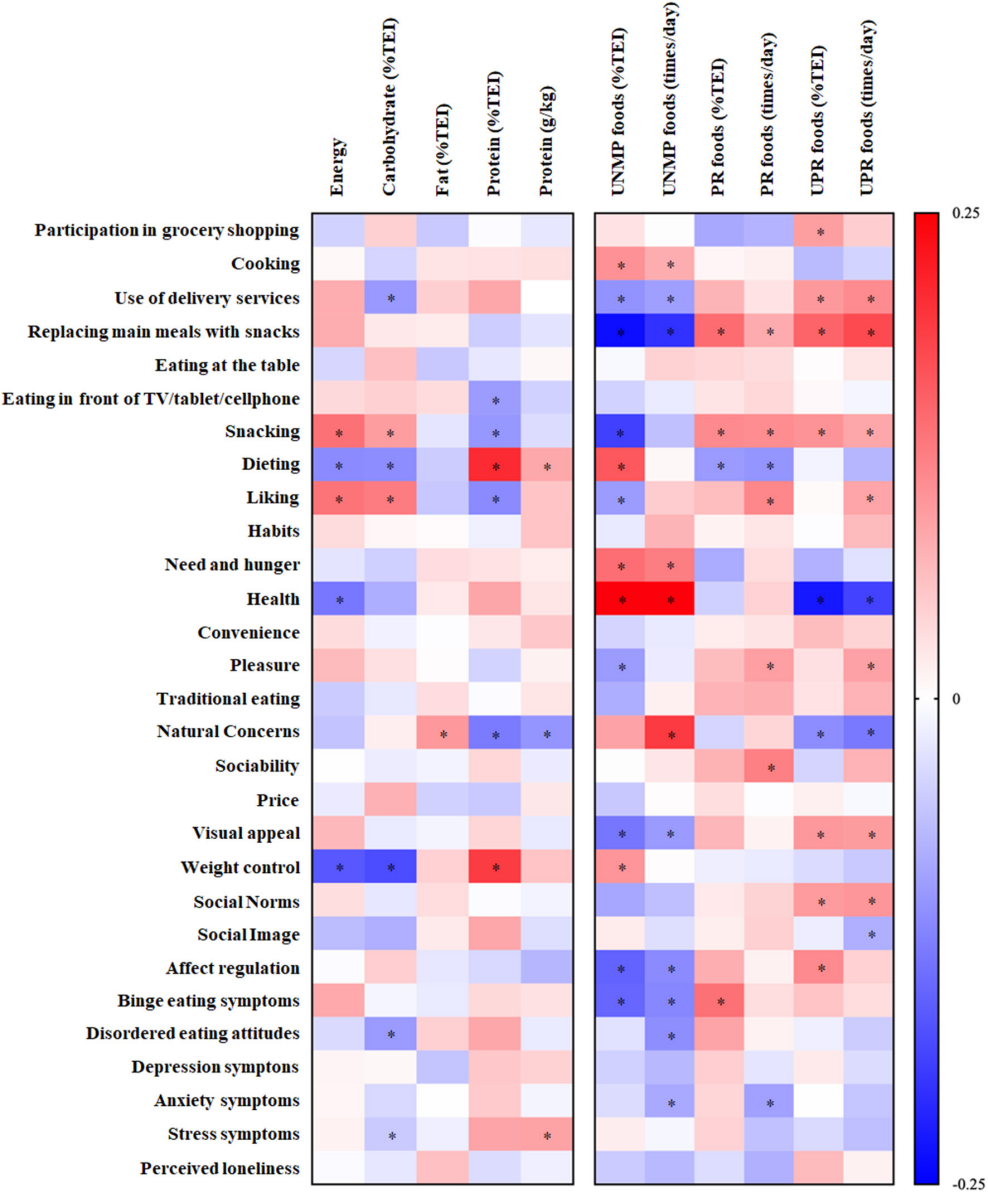
Associations between food consumption (dependent variables) and eating habits and determinants (independent variables). Data presented as standardized β (95% CI); *p < 0.05. UNMP, unprocessed and minimally processed foods; PR, processed foods; UPR, ultraprocessed foods.

“Dieting” (β = 7.27 [3.30, 11.24], *p* < 0.005), “need and hunger” (β = 3.34 [1.20, 5.49], *p* = 0.002) and “health” (β = 4.94 [3.23, 6.65], *p* < 0.0001) associated with an increased consumption of unprocessed and minimally processed foods, whereas “replacing main meals with snacks” (β = −8.98 [−12.32, −5.63], *p* < 0.0001), “snacking” (β = −6.92 [−10.14, −3.70], *p* < 0.0001) and binge eating symptoms (β = −0.34 [−0.57, −0.12], *p* = 0.002) associated with reduced consumption of foods within this processing level. In contrast, “use of delivery services” (β = 3.39 [0.37, 6.42], *p* = 0.028), “replacing main meals with snacks” (β = 5.49 [2.23, 8.76], *p* = 0.001), “visual appeal” (β = 2.17 [0.25, 4.10], *p* = 0.027), “social norms” (β = 2.19 [0.19, 4.19], *p* = 0.031) and “affect regulation” (β = 2.01 [0.35, 3.68], *p* = 0.017) associated with increased ultraprocessed food consumption (Figure 1).

Overall, visual inspection of Figure 1 indicates that the number and magnitude of associations were greater when analyzing consumption by food processing levels rather than by macronutrient intake.

**Figure 2:**
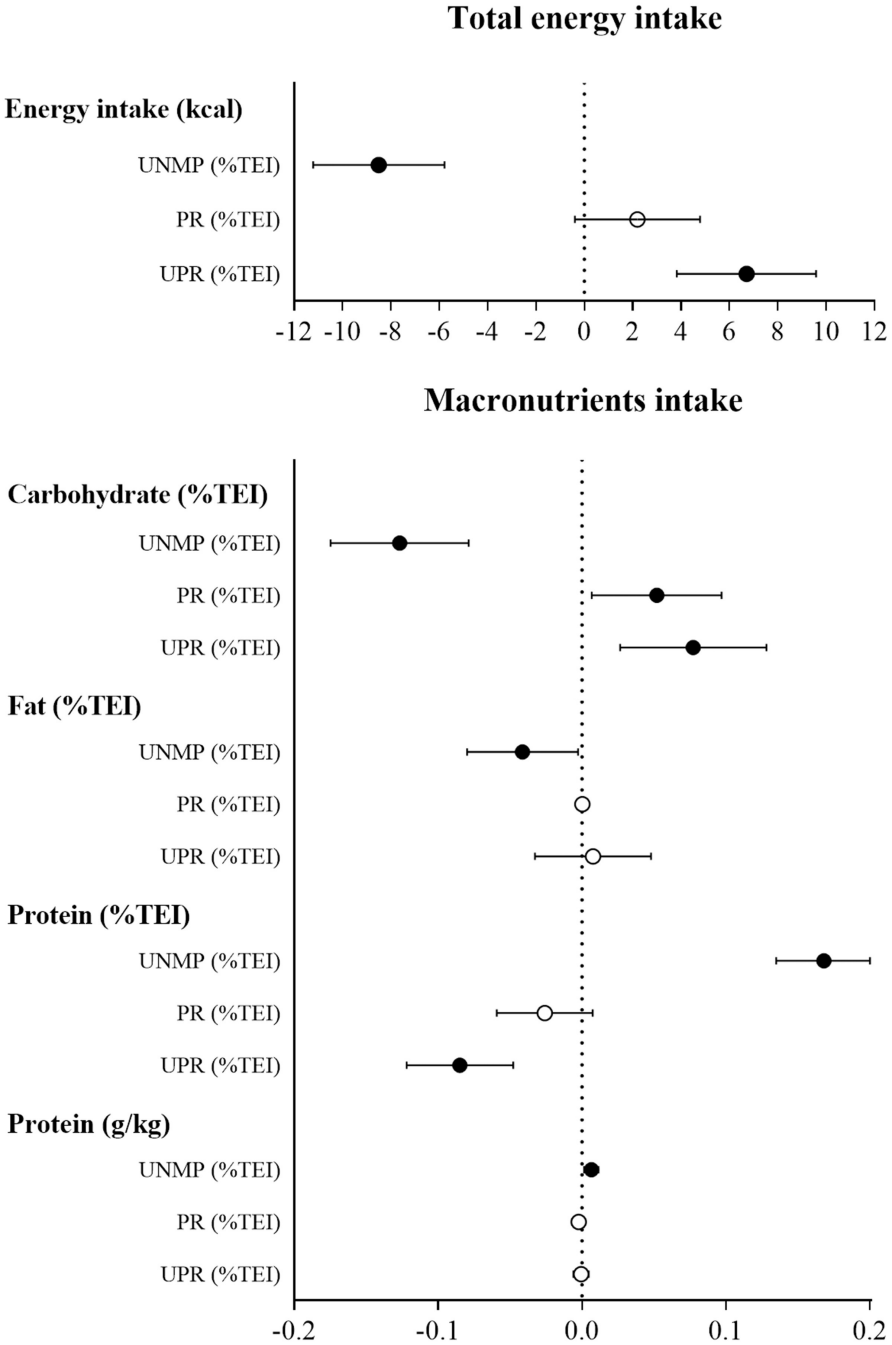
Associations between macronutrient intake (dependent variables) and food consumption by processing level (independent variables). Data presented as standardized β (95% CI); ∙*p* < 0.05; °*p* > 0.05. UNMP, unprocessed and minimally processed foods; PR, processed foods; UPR, ultraprocessed foods.

Average carbohydrate, fat, and protein intake were 45.4 [44.5, 46.2], 36.7 [36.0, 37.4], and 17.9 [17.3, 18.6] %TEI, respectively. The most prevalent food group consumed was unprocessed/minimally processed food (43.9 [42.3, 45.4] %TEI), followed by processed food (22.8 [21.2, 24.6] %TEI), ultraprocessed food (20.8 [19.3, 22.3] %TEI), and culinary ingredients (13.1 [12.5, 13.8] %TEI). Interestingly, the consumption of unprocessed or minimally processed food was associated with decreased energy (β = −8.50 [−11.21, −5.78], *p* < 0.001), carbohydrate (β = −0.13 [−0.17, −0.08], *p* < 0.001) and fat (β = −0.04 [−0.08, −0.003], *p* = 0.036) intake, and with increased protein intake (β = 0.16 [0.13, 0.20], *p* < 0.001). In contrast, the consumption of ultraprocessed food was associated with increased energy (β = 6.71 [3.83, 9.59], *p* < 0.001) and carbohydrate intake (β = 0.08 [0.03, 0.13], *p* = 0.003) and with reduced protein intake (β = −0.08 [−0.12, −0.05], *p* < 0.001). Processed food consumption was only associated with increased carbohydrate intake (β = 0.05 [0.01, 0.10], *p* = 0.024) (Figure 2).

## Discussion

The main findings of this study were that: (i) associations were stronger and more numerous when analyzing food consumption by processing level as opposed to macronutrient intake; and, more importantly, (ii) consumption of unprocessed and minimally processed foods were associated with healthier dietary patterns (e.g., food choice determined by “need and hunger” and “health”), whereas consumption of ultraprocessed foods associated with poorer dietary patterns (e.g., “snacking,” “use of delivery services” and “replacing main meals with snacks”).

In our sample, consumption of ultraprocessed foods was relatively low (~21 %TEI) when compared to other populations (range: 25.8–59.7 %TEI) (29–33). Nonetheless, a higher consumption of foods in this processing level was still associated with a nutritionally inadequate diet (i.e., higher energy and carbohydrate intake and lower protein intake), which is in line with other studies that also reported an association between ultraprocessed food consumption and less desirable dietary patterns (34–36). Particular attention should be given to high consumers of ultraprocessed foods during the COVID-19 pandemic, as this type of food is associated with increased adiposity (37–40), even in a short period of time (41).

Evidence indicates that eating habits have changed during the COVID-19 pandemic in several countries (9–14, 17, 42, 43). Increases in snacking have been consistently reported (12, 13, 17, 43), which could be attributed to an attempt to cope with the emotional stress of social isolation (44, 45). Herein, we showed that “snacking” and “replacing meals with snacks” were associated with nutritionally inadequate diets, such as those characterized by higher consumption of carbohydrate and ultraprocessed foods. In contrast, several other eating habits and food choice determinants associated with indicators of healthier dietary patterns. For instance, “dieting” and choosing foods based on “health” and “weight control” associated with a more nutritionally balanced diet. These findings are in line with the literature, which shows that people concerned with health- and weight-related matters are more prone to report a higher consumption of fruits and vegetables, and a lower consumption of red meat, snacks and sweets (18, 46, 47).

Interestingly, associations with eating habits and food choice determinants were more frequent and pronounced when analyzing food consumption by processing level rather than by macronutrient content. These findings could, at least in part, be attributed to the fact that focusing on macronutrients may result in a narrower view of food consumption as it may not fully capture the heterogeneity of eating behaviors; ultimately, people usually select what they eat based on food availability and preference rather than nutrients *per se* (48–50).

Our study is strengthened by the fact that data were collected when the most restrictive stay-at-home orders were in place, thus allowing for a more representative view of this unprecedented social context on diet. However, this study is not without limitations. Firstly, food consumption data were only analyzed for a subset of participants; however, it is important to highlight that the participants included in this study were considered representative of the total sample, as they were randomly selected, and their demographic characteristics did not significantly differ from the broader data set. Additionally, we used 1-day food diaries to assess food consumption, which may have introduced reporting bias in our study as some participants may have reported food consumption for an unusual day. Finally, our sample is predominantly composed of women with university degrees, which may limit the generalization of the present findings to other populations.

In conclusion, some eating habits and food choice determinants (e.g., “snacking,” “replacing meals with snacks” or “use of delivery services”) observed during the COVID-19 pandemic (17) associated with unhealthy dietary patterns (e.g., high energy and carbohydrate consumption, increased ultraprocessed food consumption and reduced unprocessed/minimally processed foods consumption) in Brazilian women. Interestingly, these associations were more frequent and pronounced when analyzing food consumption by processing level rather than by macronutrient content. The comprehensive understanding of the complex interplay between eating habits, food choice determinants, and food consumption may guide health professionals to identify at-risk individuals for an unhealthier diet pattern during the COVID-19 pandemic. From a practical perspective, clinical and public health interventions focused on mitigating poor eating habits (e.g., “snacking,” “replacing main meals with snacks”) should be implemented since these behaviors are associated with an increased intake of ultraprocessed foods, which, in turn, could be detrimental to overall health (34–36).

## Data Availability Statement

The raw data supporting the conclusions of this article will be made available by the authors, without undue reservation.

## Ethics Statement

The studies involving human participants were reviewed and approved by Ethics Committee for the Analysis of Research Projects of Clinical Hospital of FMUSP, Presentation Certificate for Ethical Appreciation number 33561720.2.0000.0068. The patients/participants provided their written informed consent to participate in this study.

## Author Contributions

FS and BM: conceptualization, investigation, writing-original draft, visualization, project administration, formal analysis, and funding acquisition. GE: investigation, formal analysis, writing-review and editing, and funding acquisition. HA, MA, DC, and KC: investigation, formal analysis, and writing-review and editing. FB and HR: writing-review and editing. AP: formal analysis, visualization, writing-original draft, and funding acquisition. BG: conceptualization, writing-original draft, supervision, and funding acquisition. CN: conceptualization, investigation, writing-review and editing, funding acquisition, project administration, and supervision. All authors contributed to the article and approved the submitted version.

## Conflict of Interest

The authors declare that the research was conducted in the absence of any commercial or financial relationships that could be construed as a potential conflict of interest.
